# Evidence for senescence in survival but not in reproduction in a short‐lived passerine

**DOI:** 10.1002/ece3.6281

**Published:** 2020-05-08

**Authors:** Rémi Fay, Michael Schaub, Jennifer A. Border, Ian G. Henderson, Georg Fahl, Jürgen Feulner, Petra Horch, Mathis Müller, Helmut Rebstock, Dmitry Shitikov, Davorin Tome, Matthias Vögeli, Martin U. Grüebler

**Affiliations:** ^1^ Swiss Ornithological Institute Sempach Switzerland; ^2^ British Trust for Ornithology The Nunnery Thetford UK; ^3^ Meudt Germany; ^4^ Grafengehaig Germany; ^5^ Balingen Germany; ^6^ Zoology and Ecology Department Moscow Pedagogical State University Moscow Russia; ^7^ National Institute of Biology Ljubljana Slovenia

**Keywords:** actuarial senescence, age‐specific demographic rate, aging, Saxicola rubetra, whinchat

## Abstract

Senescence has been studied since a long time by theoreticians in ecology and evolution, but empirical support in natural population has only recently been accumulating. One of the current challenges is the investigation of senescence of multiple fitness components and the study of differences between sexes. Until now, studies have been more frequently conducted on females than on males and rather in long‐lived than in short‐lived species. To reach a more fundamental understanding of the evolution of senescence, it is critical to investigate age‐specific survival and reproduction performance in both sexes and in a large range of species with contrasting life histories. In this study, we present results on patterns of age‐specific and sex‐specific variation in survival and reproduction in the whinchat *Saxicola rubetra*, a short‐lived passerine. We compiled individual‐based long‐term datasets from seven populations that were jointly analyzed within a Bayesian modeling framework. We found evidence for senescence in survival with a continuous decline after the age of 1 year, but no evidence of reproductive senescence. Furthermore, we found no clear evidence for sex effects on these patterns. We discuss these results in light of previous studies documenting senescence in short‐lived birds. We note that most of them have been conducted in populations breeding in nest boxes, and we question the potential effect of the nest boxes on the shape of age‐reproductive trajectories.

## Graphical abstract

In this article, we jointly analyzed seven datasets investigating for the first time age‐specific breeding success and survival in whinchats, a ground‐nesting passerine species. Our results supported the occurrence of senescence in survival but, in contrast to our expectations, not in breeding success. We note that previous studies documenting senescence in short‐lived birds have been conducted in populations breeding in nest boxes, and we question the potential effect of the nest boxes on the shape of age‐reproductive trajectories.

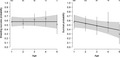

## 1 INTRODUCTION

Senescence is defined as a progressive decline in age‐specific fitness components due to internal physiological degeneration (Kirkwood & Rose, 1991). Theoreticians have extensively studied senescence over the second half of the XX century, formulated several complementary evolutionary theories, and demonstrated why it might arise under natural selection (Hamilton, 1966; Kirkwood & Rose, 1991; Medawar, 1952; Williams, 1957). Evidence for senescence in natural populations has been scarce for a long time raising doubt on the real existence of this phenomenon in the wild. However, absence of evidence mainly reflected the lack of available long‐term datasets rather than lack of senescence itself (Botkin & Miller, 1974). Over the last decades, accumulated empirical findings have shown that senescence is ubiquitous in natural animal populations affecting several fitness components (Nussey, Froy, Lemaitre, Gaillard, & Austad, 2013).

More recent studies have investigated how the rate of senescence varies between sexes and among life‐history traits (Bouwhuis & Vedder, 2017; Hayward et al., 2015). Senescence may evolve differently in the two sexes, because selection pressure could be strongly asymmetric between males and females (Bonduriansky, Maklakov, Zajitschek, & Brooks, 2008). One major theoretical prediction is that the sex experiencing higher mortality should also exhibit higher rates of senescence (Williams, 1957). When mortality due to extrinsic causes is high, there is strong selection favoring early reproduction and weak selection for survival and reproductive success later in life. Such patterns have been reported in polygynous, dimorphic ungulate species where males suffer high mortality and exhibit stronger senescence than females (Clutton‐Brock & Isvaran, 2007, but see Tidiere et al., 2015). In birds, and especially in short‐lived passerines, it is often observed that females suffer from higher mortality than males because they bear the cost associated with egg production and egg–offspring care and defense (Bennett & Owens, 2002; Donald, 2007; Promislow, Montgomerie, & Martin, 1992). Beyond the physiological cost, females are exposed to higher predation risk due to nest attendance (e.g., Low, Arlt, Eggers, & Pärt, 2010). It is therefore predicted that females show more rapid senescence than males in short‐lived passerines (Williams, 1957). Recent studies have provided some support for this prediction. For instance, house sparrow *Passer domesticus* and willow tit *Parus montanus* females have a shorter life span and higher reproductive and survival senescence rates, respectively, than males (Orell & Belda, 2002; Schroeder, Burke, Mannarelli, Dawson, & Nakagawa, 2012). In contrast, in a population of Seychelles warblers *Acrocephalus sechellensis* free from predator, survival did not differ between sexes and both sexes experience the same actuarial senescence rates (Hammers et al., 2019; Hammers, Richardson, Burke, & Komdeur, 2013).

Although comparisons of senescence patterns between sexes became more frequent in literature, they remain challenging to establish. First, the monitoring of natural animal populations is frequently biased toward females for practical reasons (Bouwhuis, Choquet, Sheldon, & Verhulst, 2012; Nussey et al., 2013). Second, obtaining descent sample sizes remains challenging especially for short‐lived species like passerines. Indeed, the only reliable method to determine the age of a bird in the wild is to mark it at a known age, which is typically only possible at the juvenile stage in many birds. A marked bird needs then to be repeatedly observed as an adult to obtain individual longitudinal data, which are mandatory to investigate senescence rates (Nussey, Coulson, Festa‐Bianchet, & Gaillard, 2008). Obtaining such data is particularly difficult for passerines due to the extensive juvenile dispersal and high mortality (Cox, Thompson, Cox, & Faaborg, 2014; Weatherhead & Forbes, 1994). Thousands of chicks need to be ringed to obtain sufficient sample size to explore age pattern in adulthood. Therefore, the vast majority of studies on senescence are conducted for a limited number of species that are common and breed in high density in nest boxes, such as tits, flycatchers, or sparrows. Yet, to fully understand the evolution of senescence, it is important to test predictions for species with contrasting ecology.

In this study, we investigate for the first time age‐specific survival and reproduction in male and female whinchat (*Saxicola rubetra*), a short‐lived ground nesting migratory passerine. To overcome the difficulty of obtaining descent sample size from known age individuals, we jointly analyzed seven individual‐based long‐term whinchat datasets. According to the evolutionary theory of senescence and previous empirical studies, we expected that (a) senescence would occur in both, survival and reproduction, and that (b) the senescence rate would be higher in females than in males due to higher mortality of females (Donald, 2007).

## 2 MATERIALS AND METHODS

### 2.1 Model species

The whinchat is a small (15 g), insectivorous Afro‐Palearctic migrant that inhabits open grasslands in both the breeding and nonbreeding areas (Cramp, 1988). Whinchats show high annual mortality, and only few individuals live more than 5 years (this study). The maximal life span ever recorded is 7 years (Fransson, Jansson, Kolehmainen, Kroon, & Wenninger, 2017). Whinchats are monogamous, nest on the ground, and usually raise one brood per year (Cramp, 1988). Individuals are sexually mature at the age of 1 year. Females incubate the clutch (typically 5–6 eggs) and brood the hatchlings, but both sexes contribute to feeding the nestlings (Cramp, 1988). Similar to most farmland birds, whinchats have undergone massive population declines over the last decades (86% between 1980 and 2016 at the European scale, IUCN, 2019) due to agricultural intensification.

### 2.2 Data collection

This study is based on the long‐term monitoring of seven whinchat breeding populations from five European countries (United Kingdom 1, Slovenia 1, Russia 1, Germany 3, Switzerland 1). The duration of data sampling varied from 5 to 16 years depending on the population (Table 1). These populations are too far away from each other to allow substantial exchange among them and are thus demographically independent. In each population, both adults (1 year old or older) and nestlings were ringed with aluminum and color plastic rings. Based on plumage characteristics, some adults that were captured for the first time could be aged as 1 year old. Although the sex of nestlings was unknown, sex was systematically recorded for birds ringed or reobserved as adults based on plumage dimorphism. In total, 5,553 individuals have been ringed. A substantial proportion of them, however, have been ringed as fledgling and were never observed thereafter. Only 6% of all ringed nestlings have been recaptured as adults, which is a standard return rate in passerines (Weatherhead & Forbes, 1994). In the end, the life histories of 1,461 adults have been recorded, and the exact age was known for 493 of them. Additionally, whinchat nests were searched and the presence of fledglings was recorded to determine the breeding success. A marked individual was considered successful if it had produced at least one fledgling during the breeding season. The number of nest visits was kept at a minimum in order to reduce the potentially negative impact of the monitoring. The available information about reproduction was therefore limited to whether or not a brood was successful (produced at least one fledgling).

**TABLE 1:** Location, duration of data sampling and sample sizes for the seven whinchat populations

Population	Salisbury Plain	Ljubljanska barje	Topornya	Balingen	Westerwald	Oberfranken	Engadine valley
Location and study duration							
Country	United Kingdom	Slovenia	Russia	Germany	Germany	Germany	Switzerland
Monitoring period	2010–2014	2002–2014	2001–2016	1983–1993	1979–1984	1990–1994	1989–1993
Breeding success (number of broods)							
Known age	91	41	276	25	20	24	0
Unknown age	70	341	245	147	70	92	0
Total	161	382	521	172	90	116	0
Ringed individuals							
Nestlings (recaptured as adults)	292 (40)	1,066 (35)	1601 (22)	372 (26)	495 (18)	233 (9)	219 (36)
One year old	41	9	252	1	0	4	0
Unknown age	63	289	296	136	63	73	48
Total	396	1,364	2,149	509	558	310	267

### 2.3 Data analysis

All capture–recapture datasets were analyzed jointly with a Cormack–Jolly–Seber (CJS) model using the state‐space likelihood fitted in the Bayesian framework (Kéry & Schaub, 2012). We fitted two models to the data: the first model allowed for sex‐specific senescence rates while the second model assumes a common senescence rate and a sex‐specific intercept. Parameters directly estimated by the model were φ_i,j,t_, the apparent survival probability of individual *i* in population *j* in year *t*, and p_i,j,t_, the recapture probability (Lebreton, Burnham, Clobert, & Anderson, 1992). We applied the following linear model for the survival probabilities of the known‐aged individuals:

(1) $$\text{logit}\left(\phi_{i,j,t}\right)\;=\;\alpha_{{\text{age}}_{i,t},{\text{sex}}_{i}}\;+\;\varepsilon_{j}^{\text{pop}}\;+\;\varepsilon_{i}^{\text{ind}}$$

where $\varepsilon_{j}^{\text{pop}}\;∼\;N\left(0,\sigma_{\mathrm{pop}}^{2}\right)$ and $\varepsilon_{i}^{\text{ind}}\;∼\;N\left(0,\sigma_{\text{ind}}^{2}\right)$ . Age_i,t_ and sex_i_ are categorical explanatory variables indicating for each individual *i* and year *t* the current age and the sex, respectively, and *α_a,s_* is the estimated annual survival of individuals of age *a* and sex *s* on the logit scale. The individual random effect ( $\varepsilon_{i}^{\text{ind}}$ ) accounts for the nonindependence among multiple observations over an individual's life history and for the among‐individual heterogeneity. The adults of unknown age were jointly analyzed to improve the estimation of random effects. Individuals of unknown age were modeled with the following linear model:

$$\text{logit}\left(\Phi_{i,j,t}\right)\;=\;\gamma \;+\;\beta_{j,\text{se}x_{i}}\;+\;\varepsilon_{j}^{\text{pop}}\;+\;\varepsilon_{i}^{\text{ind}}$$

Here, γ is the mean logit survival of females, *β_p_* is the difference of survival between sexes in population *p*, and $\varepsilon_{j}^{\text{pop}}$ and $\varepsilon_{i}^{\text{ind}}$ are the same random effects (variability among populations and variability among individuals) as for the known‐aged individuals (Equation 1). The population‐specific difference in survival between sexes was modeled with a further random effect as $\beta_{p}\;∼\;N\;\left(d,\sigma_{d}^{2}\right)$ , where *d* is the mean difference and $\sigma_{d}^{2}$ the variability of the differences among populations. The recapture probabilities for both the individuals of known and of unknown age were modeled as $\log\text{it}\left(p_{i,j,t}\right)\;=\;\eta_{j,{\text{sex}}_{i}}\;+\;\varepsilon_{t,j}^{p}$ , where $\eta_{p,s}$ is the mean logit recapture probability of individuals in population *p* and of sex *s*, and $\varepsilon_{t,j}^{p}\;∼\;N\left(0,\sigma_{p}^{2}\right)$ . $\sigma_{p}^{2}$ is the population‐specific temporal variability in recapture probability.

The second model that was fitted to the same data assumed that the age effect on survival is the same in both sexes; thus, there is no interaction anymore between age and sex. Consequently, Equation 1 is replaced by:

(2) $$\text{logit}\left(\Phi_{i,j,t}\right)\;=\;\alpha_{{\text{age}}_{i,t}}^{{}^{\prime }}\;+\;\beta_{j,{\text{sex}}_{i}}\;+\;\varepsilon_{j}^{\text{pop}}\;+\;\varepsilon_{i}^{\text{ind}}$$

where $\alpha_{a}^{{}^{\prime }}$ is the annual logit survival of individuals of age *a*. The other parameters are the same as explained above, and also, the model parts for the individuals of unknown age and for the recapture probabilities are the same as explained above. The tow analyses were restricted to individuals that survived to adulthood (age 1) treating the first breeding observation as the year of initial marking (Pradel, Hines, Lebreton, & Nichols, 1997). By doing this, we excluded the survival estimates from fledging to age 1, which include high rates of natal dispersal and preclude a direct comparison with survival estimated at older age (Weatherhead & Forbes, 1994).

Breeding success of individuals of known age was analyzed using the following generalized linear mixed model.

$${\text{BS}}_{i,j,t}\;∼\;\text{Bern}\;\left(\omega_{i,j,t}\right)$$

(3) $$\text{logit}\left(\omega_{i,j,t}\right)\;=\;\alpha_{{\text{age}}_{i,t}}\;+\;\gamma_{j}^{\text{pop}}\;+\;\gamma_{t}^{\text{year}}\;+\;\gamma_{i}^{\text{ind}}$$

where $\gamma_{j}^{\text{pop}}\;∼\;N\;\left(0,\sigma_{\text{pop}}^{2}\right)$ , $\gamma_{t}^{\text{year}}\;∼\;N\;\left(0,\;\sigma_{\text{year}}^{2}\right)$ and $\gamma_{i}^{\text{ind}}\;∼\;N\;\left(0,\sigma_{\text{ind}}^{2}\right)$ . Age_i,t_ is a categorical explanatory variable indicating for each individual *i* and year *t* the current age, and *α_a_* is the estimated annual breeding success of individuals of age *a* on the logit scale. $\sigma_{\text{pop}}^{2}$ , $\sigma_{\text{year}}^{2}$ and $\sigma_{\text{ind}}^{2}$ describe variability among populations, across time and individuals, respectively. As for the survival analyses, the adults of unknown age were jointly analyzed to improve the estimation of random effects. For the analysis of the breeding success of individuals of unknown age, we replaced Equation 3 by.

(4) $$\text{logit}\left(\omega_{i,j,t}\right)\;=\;\mu \;+\;\gamma_{j}^{\text{pop}}\;+\;\gamma_{t}^{\text{year}}\;+\;\gamma_{i}^{\text{ind}}$$

where *μ* is the mean breeding success on the logit scale. All the other parameters are shared with those from Equation 3. We were not able to control for the age of the breeding partner because of the extremely low number of pairs with both individuals of known age.

Senescence in survival and reproduction were assessed by fitting a linear model on the age‐specific mean posterior estimates. These regressions were done based on 12,000 replicates extracted from the posterior distributions to compute credible intervals. Models were fitted with a Bayesian approach using Markov Chain Monte Carlo (MCMC) techniques and run in software JAGS (Plummer, 2003). We used vague normal priors for the regression coefficients and uniform priors for the standard deviations of the random effects. Posterior summaries from three MCMC chains were based on 100,000 iterations after a burn‐in of 20,000 and a thinning of 10. We confirmed parameter convergence using the Gelman–Rubin statistic. All R‐hat values were below 1.1 indicating convergence of the Markov chains. We report posterior means, their associated 95% credible intervals and the probabilities that the estimates were higher or lower than zero. The goodness of fit of the capture–recapture models was assessed with the program U‐CARE (Choquet, Lebreton, Gimenez, Reboulet, & Pradel, 2009). Results are presented in Appendix S1.

## 3 RESULTS

**FIGURE 1:**
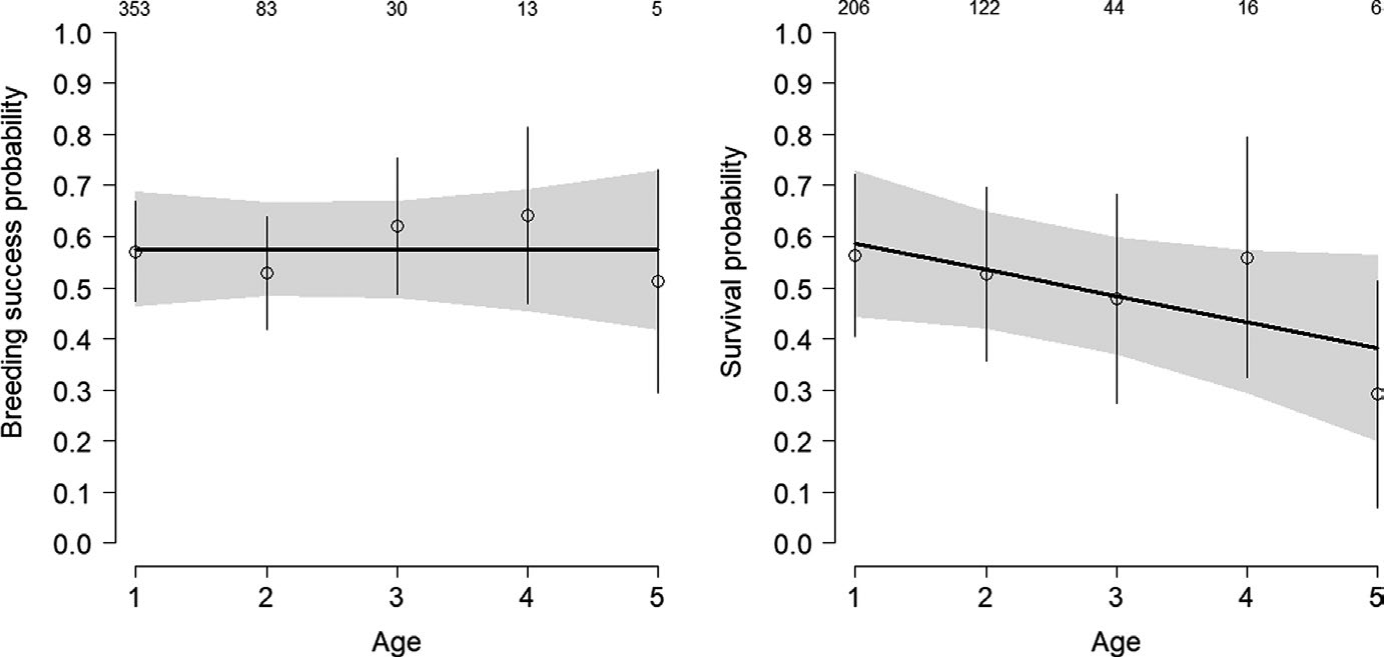
Age‐specific breeding success and survival in whinchats (both sexes combined). The lines represent the predicted relationship obtained from the linear models. The open dots show the age‐specific posterior means for breeding success and survival. The gray‐shaded area and the vertical lines represent standard errors. Sample sizes are placed over the dots. The graphs show the estimates from the UK population. The estimates for all other populations show the same patterns with age, but with a shift in the intercept

**FIGURE 2:**
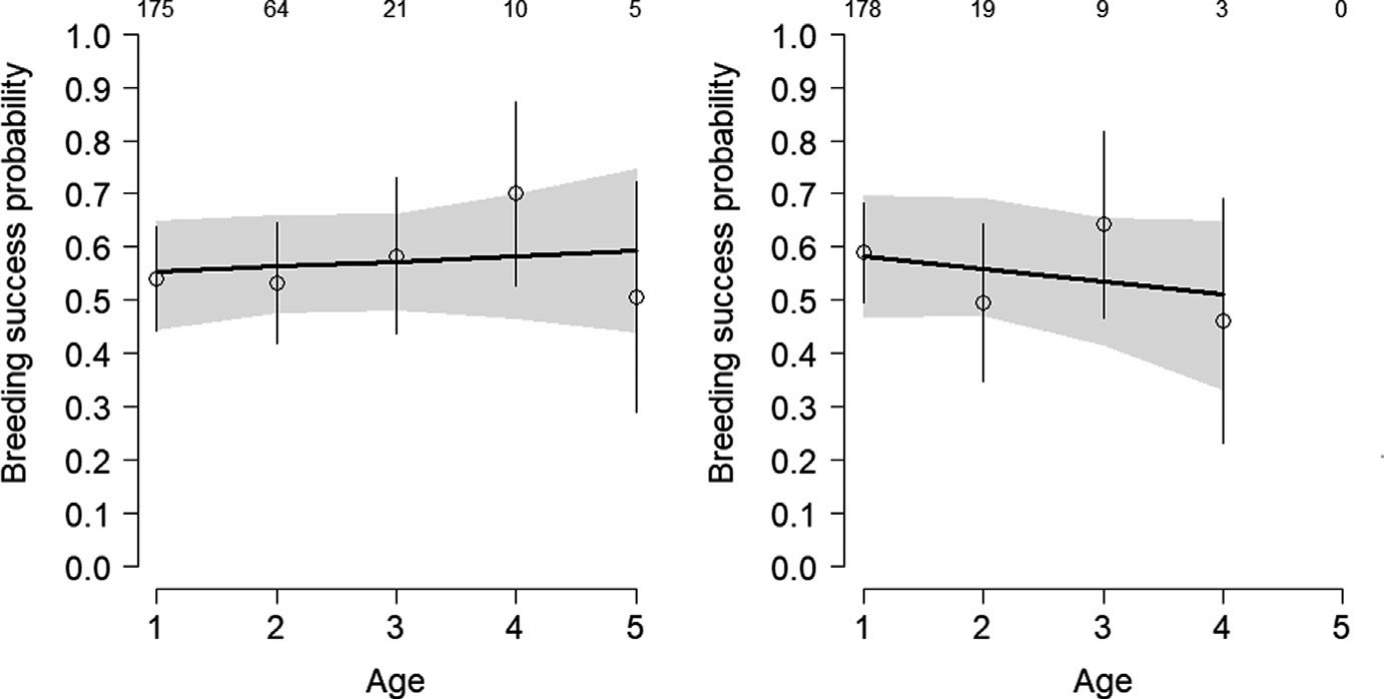
Male (left panel) and female (right panel) age‐specific breeding success in the whinchat. The plain lines represent the predicted relationships obtained from linear models. The open dots show the age‐specific posterior means. The gray‐shaded areas and the vertical lines represent ± standard errors. Sample sizes are placed over the dots

**FIGURE 3:**
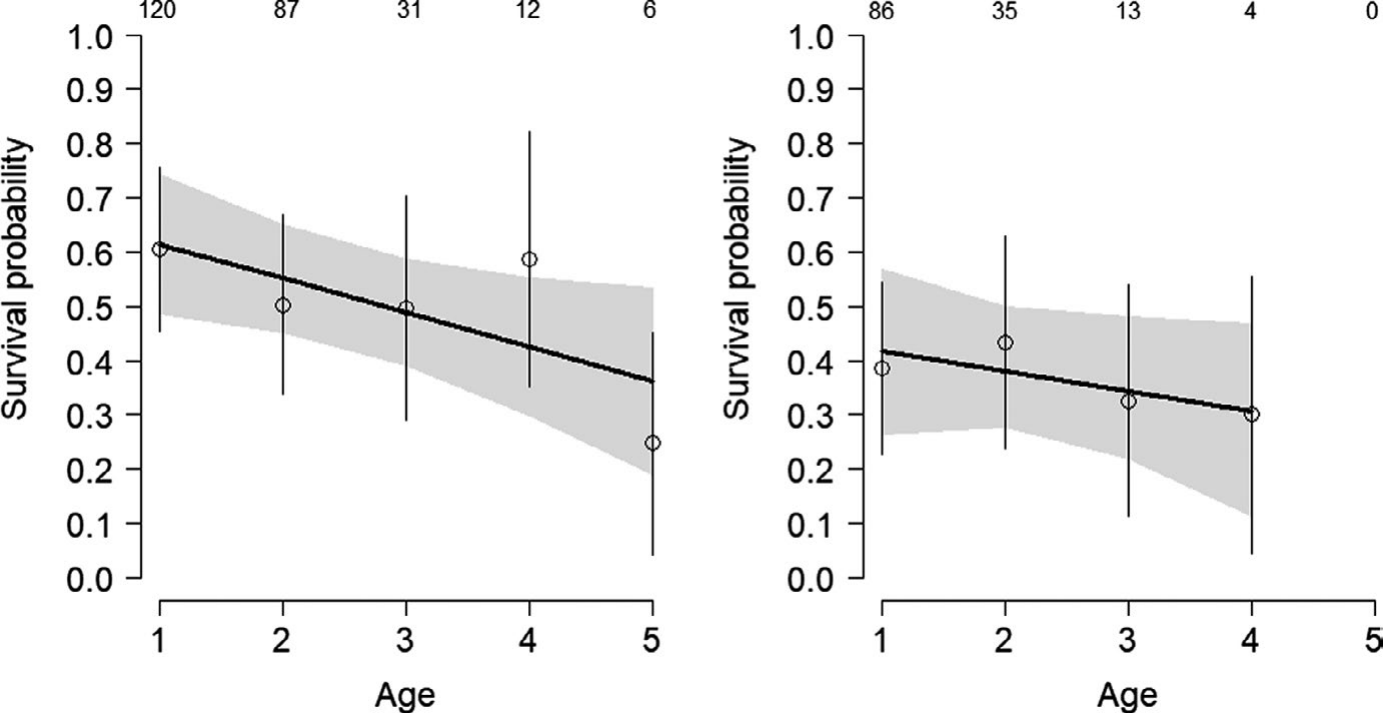
Male (left panel) and female (right panel) age‐specific annual survival in the whinchat. The plain lines represent the predicted relationships obtained from linear models. The open dots show the age‐specific posterior means. The gray‐shaded area and the vertical lines represent ± standard errors. Sample sizes are placed over the dots

The average recapture probabilities were lower in females (.49) than in males (.66), and varied among populations from .15 to .65 and .56 to .81 for females and males respectively. The estimated sex effect on average survival was positive (*d* = .53[−0.01, 1.05], *p*(*d*)>0 = .97), strongly suggesting higher survival in males than in females. The results suggested that survival declined with increasing age (−.05 [−.16, .07], *p* < 0 = .81). The estimated decline in survival from age 1 to 5 was roughly 10% per year (Figure 1). When estimating sex‐specific actuarial senescence, the survival decline seemed clearer in males than in females (*p*(slope)<0 = .86 and .68 respectively, Figure 3), but this may be due to the lower sample size in females. Available data provided weak evidence for sex‐specific actuarial senescence. The probability that the survival decrease was stronger for males than for females was only .60. The average annual breeding success across all populations and years was .79 [.77, .81]. Breeding success did not decline with increasing age, neither overall (.00 [−.22, .36], *p* < 0 = .49, Table 2, Figure 1) nor when the sex was accounting for (*p* < 0 = .42 and 62, Figure 2). These results suggest that, in contrast to survival, breeding success remained constant across the life span in both sexes.

**TABLE 2:** Posterior means and 95% credible intervals of the slopes of the regression of breeding success and survival against age in the whinchat. Given are also the probabilities that the estimates are positive. Sample sizes are given on Figures 1, 2, 3

	Mean (slope)	95% CRI	*p*(slope)<0
Breeding success			
Male	0.010	[−0.084, 0.105]	.42
Female	−0.024	[−0.160, 0.123]	.62
Both	−0.001	[−0.093, 0.094]	.51
Survival			
Male	−0.063	[−0.169, 0.059]	.86
Female	−0.037	[−0.198, 0.166]	.68
Both	−0.051	[−0.159, 0.070]	.81

## 4 DISCUSSION

Age‐specific and sex‐specific trajectories in short‐lived species like passerines are highly challenging to investigate and thus, they are currently poorly known. Here, we were able to provide new insights in this research area by jointly analyzing datasets on breeding success and survival from seven whinchat populations. Our results supported the occurrence of senescence in survival but, in contrast to our expectations, not in breeding success. Moreover, sex differences in age–trajectories were weak if existing at all. Despite uncertainty in slope estimates, our raw estimates and average effect sizes suggest clear patterns. While breeding success seemed independent of age, survival declined substantially with increasing age with a biologically relevant effect size.

Survival decreased monotonously from age 1 to 5 without evidence of a difference between sexes in the whinchat. Such a continuous decline of survival matches the average‐age survival trajectory described in song sparrows (*Melospiza media*) for both sexes (Keller, Reid, & Arcese, 2008). Characteristically, survival in short‐lived passerines decreases continuously after a maximum value that is reached early in life (Balbontín & Møller, 2015; Bouwhuis et al., 2012; Orell & Belda, 2002; Sendecka, 2007; Sternberg, 1989). The rapid decline of survival in passerines is in line with our evolutionary understanding of senescence in the framework of life‐history theory. In accordance with predictions, senescence has been shown to occur proportionally earlier and stronger in species with increasing speed of life (Jones et al., 2008; Péron, Gimenez, Charmantier, Gaillard, & Crochet, 2010).

Whereas higher natural mortality rates in females are widely expected in passerines (Donald, 2007), there is only limited evidence for sex differences in actuarial senescence rates from the wild. The few studies investigating survival senescence in both sexes of passerines provide generally no evidence of differences between sexes (Balbontín & Møller, 2015; Hammers et al., 2013; Sendecka, 2007; Sternberg, 1989). However, when a sex effect was present, senescence was stronger in females (Orell & Belda, 2002). The difference between theory and empirical studies could reflect the overestimation of the expected female mortality due to the confounding effect of dispersal (Dale, 2001). Alternatively it could also reflect the difficulty to detect small sex effects in senescence due to low statistical power (Nussey et al., 2008). The absence of evidence is not evidence of absence (Altman & Bland, 1995). In our case the modest sample sizes especially for females did not allow for a definitive conclusion.

Surprisingly, we found no support for reproductive senescence. This is unexpected because reproductive decline at old age seems to be ubiquitous in animal populations (Lemaître & Gaillard, 2017; Nussey et al., 2013). Although the onset of reproductive senescence may be slightly delayed by the progressive improvement of competence in early life, studies examining survival and reproductive senescence in passerine observed generally a decrease in both fitness components (Bouwhuis et al., 2012; Keller et al., 2008; Sendecka, 2007). It is unlikely that the apparent absence of reproductive senescence was a consequence of lack of statistical power or of most whinchats not having reached the age at the onset of senescence. In fact, only very few whinchat individuals are assumed to exceed the age of 5 years and survival decreased continuously from the age of 1 year. Rather the metric used for reproductive performance, that is, breeding success based on the presence of at least one fledgling, may have prevented the detection of a decline in reproduction at old ages. Future studies should use other components of productivity such as clutch size or the number of fledglings, information that, unfortunately, was not available in our case. The whinchat breeding ecology is another important aspect to consider in relation to the apparent absence of reproductive senescence. It is well known that the nesting site is a key factor to understand the life‐history traits of birds (Martin & Li, 1992). The whinchat as a ground‐breeding species in open landscapes is exposed to a high predation risk and other environmental perturbations making breeding success fairly random. This may be accentuated in agricultural landscape by mowing activities, which lead to nest destruction. This high stochasticity may impede the detection of an age‐related decline.

Interestingly, most previous studies documenting reproductive senescence in passerines have been carried out in populations breeding in nest boxes (tits, flycatchers, sparrows, swallows). Nest boxes are known to positively affect the breeding success because the exposition to predators and weather is reduced (Fay, Michler, Laesser, & Schaub, 2019; Møller, 1989). Furthermore, researchers have regularly implemented measures to reduce predation on their studied breeding population. For instance, the nest boxes of the famous Great tit population in the Wytham Wood (Oxfordshire) have been made predator proof since the 1970s. Before, weasel predation reached up to 50% in some years (McCleery, Clobert, Julliard, & Perrins, 1996). The stochasticity introduced by predation on breeding success would have stronger negative effects on age–classes with high performance just because they have more to lose. For instance, if we assume random predation with respect to age, a predation risk of 50% would have a stronger negative effect on an average breeding success of 0.8 (average loss of 0.4) than on an average breeding success of 0.4 (average loss of 0.2). Thus, we may wonder if the reproductive senescence occurring in natural populations may have been overestimated in these nest‐box populations due to a decrease of random perturbations. If true, this means that the strength of natural selection against senescence is lower than expected. Clearly, the potential effect of nest boxes on the shape of observed age‐reproductive trajectories has been overlooked until now and requires further attention.

As in most demographic studies, survival estimated from capture–recapture data includes permanent emigration. Although dispersal is much more important at the juvenile stage, adults, and especially adult females, may still disperse substantially (Clarke, Sæther, Røskaft, Saether, & Roskaft, 1997). Thus, the higher survival of whinchat males may partly be due to sex‐specific differences in dispersal (Bastian, 1992). This raises the question whether the detected senescence effect on apparent survival is in fact caused by increasing dispersal. To the best of our knowledge, increasing dispersal with increasing age has never been documented in passerines. It seems more reasonable to assume that breeding dispersal is age‐independent, and the decline in apparent survival is a consequence of senescence in true survival. Consistently, the strongly philopatric whinchat males show survival estimates that clearly decrease with age, similarly with the average pattern reported in Figure 1.

To conclude, this study contributes to our understanding of senescence in natural populations showing for the first time evidence of actuarial senescence in the whinchat. Surprisingly we found no evidence of reproductive senescence and speculate whether stochastic factor such as predation may have contributed to this result. We hope that this study will stimulate more empirical studies in a wider range of species.

## CONFLICT OF INTEREST

None declared.

## AUTHOR CONTRIBUTIONS

**Rémi Fay:** Conceptualization (lead); Formal analysis (lead); Methodology (lead); Writing‐original draft (lead); Writing‐review & editing (lead). **Michael Schaub:** Formal analysis (supporting); Methodology (supporting); Writing‐review & editing (equal). **Jennifer A. Border:** Data curation (equal); Resources (equal); Writing‐review & editing (equal). **Ian G. Henderson:** Data curation (equal); Resources (equal); Writing‐review & editing (equal). **Georg Fahl:** Data curation (equal); Resources (equal); Writing‐review & editing (equal). **Jürgen Feulner:** Data curation (equal); Resources (equal); Writing‐review & editing (equal). **Petra Horch:** Data curation (equal); Resources (equal); Writing‐review & editing (equal). **Mathis Müller:** Data curation (equal); Resources (equal); Writing‐review & editing (equal). **Helmut Rebstock:** Data curation (equal); Resources (equal); Writing‐review & editing (equal). **Dmitry Shitikov:** Data curation (equal); Resources (equal); Writing‐review & editing (equal). **Davorin Tome:** Data curation (equal); Resources (equal); Writing‐review & editing (equal). **Matthias Vögeli:** Data curation (equal); Resources (equal); Writing‐review & editing (equal). **Martin U. Grüebler:** Resources (equal); Supervision (equal); Writing‐review & editing (equal).

## Supporting information

Supplementary Material

## Data Availability

Dryad deposit: https://datadryad.org/stash/share/oW1kRAv6‐plMQHZpVciayCJ0fd50m4pOFEJpy‐lBlAo
