# Supplementary material for: Evidence for senescence in survival but not in reproduction in a short‐lived passerine
Source: Ecol Evol. 2020 May 8;10(12):5383–90. doi: 10.1002/ece3.6281 (PMC7319115; doi:10.1002/ece3.6281)
Supplement: Supplementary file 1 — Supplementary Material [file ECE3-10-5383-s001.docx]

**Appendix S1** : Goodness of Fit

Goodness-of-fit (GOF) tests for Cormack-Jolly-Seber model were performed with U-CARE (Choquet et al. 2009). We removed the first capture occasion of birds ringed as nestlings to account for an age effect. Due to the sparseness of some datasets GOF tests have been performed for models without a sex effect.

|  | Overall test | | |
| --- | --- | --- | --- |
| Population | χ^2^ | df | p-value |
| UK | 3.61 | 7 | 0.82 |
| SL | 25.31 | 27 | 0.56 |
| RU | 28.22 | 20 | 0.10 |
| Ba | 14.29 | 15 | 0.50 |
| We | 0.78 | 5 | 0.98 |
| Ob | 0 | 5 | 1 |
| Sw | 0.60 | 3 | 0.90 |

The general CJS model fitted adequately for most of the datasets (p-value≥0.5). However, the GOF test for the Russian (RU) population was less satisfactory (p-value=0.1). A closer examination showed that, contrary to other datasets, some assumptions were not met for this population (see table below). The significant 3.SR test suggested the presence of transients. This is problematic, because transients negatively bias survival estimates. Thus, we controlled for transients in the Russian population by adapting our models accordingly (Pradel 1997). For this purpose, we explicitly estimated different survival probabilities of newly marked and of previously marked individuals. We then only used the latter estimate for making inference about survival.

| Population | RU | | |
| --- | --- | --- | --- |
|  | χ^2^ | df | p-value |
| TEST3.SR | **27.33** | **12** | **0.01** |
| TEST3.SM | 0.89 | 5 | 0.97 |
| TEST2.CT | 0.00 | 3 | 1 |
| TEST2.CL | NA | NA | NA |

References

Choquet, R., Lebreton, J.-D., Gimenez, O., Reboulet, A.-M., Pradel, R., 2009. U-CARE: Utilities for performing goodness of fit tests and manipulating CApture–REcapture data. Ecography 32, 1071-1074.

Pradel, R., Hines, J.E., Lebreton, J.D., Nichols, J.D. 1997. Capture-recapture survival models taking account of transients. Biometrics, 53, 60-72.
